# HOUSING AFFORDABILITY AND INTER-REGIONAL MOVES AMONG OLDER ADULTS

**DOI:** 10.1093/geroni/igz038.1023

**Published:** 2019-11-08

**Authors:** Sarah Mawhorter, Jennifer A Ailshire

**Affiliations:** University of Southern California, Los Angeles, California, United States

## Abstract

Housing prices have risen in urban areas across the US since 2000, with only a brief interruption after the housing crisis of 2008. At the same time, prosperous urban areas have pulled away from declining urban and rural areas. Older adults are more likely to be affected by both increases and divergence of housing prices: owners may not be able to afford rising property taxes (though they benefit from increasing home equity), and renters are especially vulnerable. Housing affordability constraints may also affect the places where older adults can afford to move. In this paper, we compare the residential mobility patterns of adults aged 50+ living in high-cost, mid-cost, and low-cost areas from 2000-2014, using data from the Health and Retirement Study with county-level US Census and American Community Survey contextual data, as well as the Zillow Home Value Index. We find that both homeowners and renters living in high-cost areas remain in place at higher rates compared with those living in mid-cost and low-cost areas. Among those who move, older adults living in high-cost regions move towards mid-cost and low-cost regions more often than the reverse. The differences are particularly pronounced for renters. The overall outcome is a net movement of older adults away from high-cost areas towards mid-cost and low-cost areas. These shifts have consequences for the well-being of older adults facing budget constraints that may limit the areas where they can afford to live or move, and broader implications for the future of urban areas.

